# Dietary patterns and their associations with overweight/obesity among preschool children in Dongcheng District of Beijing: a cross-sectional study

**DOI:** 10.1186/s12889-021-10240-x

**Published:** 2021-01-27

**Authors:** Kaiyuan MIN, Jing WANG, Wei LIAO, Thomas Astell-Burt, Xiaoqi FENG, Shuya CAI, Yang LIU, Peiwen ZHANG, Fenghua SU, Kexin YANG, Liang SUN, Juan ZHANG, Lianjun WANG, Zechen LIU, Yu JIANG

**Affiliations:** 1 School of Population Medicine and Public Health, Chinese Academy of Medical Sciences/Peking Union Medical College, Dongcheng District, Beijing, China; 2 Institute of Basic Medical Sciences, Chinese Academy of Medical Sciences/Peking Union Medical College, Dongcheng District, Beijing, China; 3 Dongcheng Center for Disease Control and Prevention, Dongcheng District, Beijing, China; 4 Department of Epidemiology and Biostatistics, Institute of Basic Medical Sciences, Chinese Academy of Medical Sciences/Peking Union Medical College, Dongcheng District, Beijing, China; 5 Population Wellbeing and Environment Research Lab, School of Health and Society, Faculty of Arts, Social Sciences, and Humanities, University of Wollongong, Wollongong, Australia; 6 National Institute of Environmental Health, Chinese Center for Disease Control and Prevention, Beijing, China; 7 Menzies Centre for Health Policy, University of Sydney, Sydney, Australia; 8 School of Population Health, Faculty of Medicine and Health, University of New South Wales, Sydney, Australia; 9 National Institute for Nutrition and Health, Chinese Center for Disease Control and Prevention, Dongcheng District, Beijing, China; 10 School of Health Policy and Management, Chinese Academy of Medical Sciences/Peking Union Medical College, Dongcheng District, Beijing, China; 11 Department of Epidemiology, Harvard T.H. Chan School of Public Health, Boston, MA USA

**Keywords:** Dietary patterns, Overweight, Obesity, Preschool children

## Abstract

**Background:**

Few studies investigated the associations between dietary patterns and overweight/obesity among Chinese preschool children. Thus, the study aims to explore dietary patterns and their associations with overweight/obesity among preschool children in the Dongcheng District of Beijing.

**Methods:**

With a stratified proportionate cluster sampling, the study included 3373 pairs of preschool children and their guardians. Children’s weight and height were measured by school nurses, and their food and beverage consumption frequencies were reported by guardians via a food frequency questionnaire. Children’s age, gender, physical activity time, and sedentary time, as well as their parents’ highest level of educational attainment, occupation, weight, and height were also collected. Dietary patterns were identified through exploratory factor analysis. Among these identified dietary patterns, the one with the largest factor score was defined as the predominant dietary pattern for each child. The associations between predominant dietary patterns and overweight/obesity were tested by two-level random-intercept logistic models with cluster-robust standard errors.

**Results:**

Four dietary patterns, i.e., a “Sugar-sweetened beverage (SSB) and snack” pattern, a “Chinese traditional” pattern, a “Health conscious” pattern, and a “Snack” pattern, were identified. Among the children, 21.02% (95% *CI*: 19.68 to 22.43%) were predominated by the “SSB and snack” pattern, 27.78% (95% *CI*: 26.29 to 29.32%) by the “Chinese traditional” pattern, 24.90% (95% *CI*: 23.47 to 26.39%) by the “Health conscious” pattern, and 26.30% (95% *CI*: 24.84 to 27.81%) by the “Snack” pattern. After controlling for potential confounders, the “SSB and snack” pattern characterized by fresh fruit/vegetable juice, flavored milk drinks, carbonated drinks, flavored fruit/vegetable drinks, tea drinks, plant-protein drinks, puffed foods, fried foods, and Western fast foods was associated with a higher risk of overweight/obesity (*OR*: 1.61, 95% *CI*:1.09 to 2.38), compared with the “Chinese traditional” pattern.

**Conclusions:**

The preference for dietary patterns with high energy density but low nutritional value was prevalent among preschool children in the Dongcheng District of Beijing. Comprehensive measures to simultaneously reduce consumption of SSBs and unhealthy snacks among preschool children should be taken urgently to address the childhood obesity problem in China, particularly in metropolises.

**Supplementary Information:**

The online version contains supplementary material available at 10.1186/s12889-021-10240-x.

## Background

Maintaining a healthy weight status in childhood is important, given that childhood obesity can increase the risk of short- and long-term adverse consequences, both physically and mentally [1]. It’s worrying that the prevalence of overweight and obesity among children and adolescents has increased all over the world [2], and very young children were affected — about forty-one million children aged under five were overweight or obese in 2016 globally, with almost half of them living in Asia [3]. Although there appears to be a plateau of the obesity epidemic among children and adolescents in some developed countries [4], the prevalence of obesity among children and adolescents aged seven to eighteen increased from 0.1 to 7.3% in China from 1985 to 2014 [5]. Besides, inequalities in the prevalence of overweight and obesity were documented in China — a greater prevalence increase was observed among higher socioeconomic status (SES) children [6]. For example, in Beijing, the capital city of China, the prevalence of overweight and obesity among preschool children (19.44% in 2016 in Shijingshan District) [7] was comparable to some developed countries where the rate was estimated to be 11.7% in 2010 and expected to reach 12.9% in 2015 [8].

Poor eating habits are increasingly contributing to the surging global burden of non-communicable diseases [9]. Eating habits in the early years could probably track to childhood and form the basis of eating patterns in adulthood [10, 11]. In China, where people’s dietary patterns have changed significantly in the past four decades along with the rapid economic development [12], children have been exposed to unhealthy food environments [13] which would potentially facilitate unhealthy dietary patterns. For example, China is Coke’s third-largest market by volume [14], and the consumption of sugar-sweetened beverages (SSBs) has become popular among children and adolescents [15]. There are plenty of studies focused on dietary patterns among preschool children [16–23], with several of them conducted in China [17, 18, 22, 23]. However, few investigated the associations between dietary patterns and overweight/obesity among Chinese preschool children [23]. While the prevalence of childhood obesity has significantly increased in recent years in China [5], the association with dietary patterns is still unclear. Therefore, the present study aims to identify dietary patterns and examine their associations with overweight/obesity among preschool children in the Dongcheng District of Beijing.

## Methods

### Study design and participants

#### Study settings

The study was conducted in local kindergartens, that is, schools that provide normal education to children aged three to six in the Dongcheng District, the eastern half of the downtown area of Beijing. The study was approved by the Ethics Committee of Dongcheng Center for Disease Control and Prevention (DCCDPCIRB-20180416-1).

#### Eligibility criteria

As children in the third year of kindergarten are going to attend primary schools, eligible participants were children registering in the first and second years of chosen kindergartens at the time of recruitment. As many preschool children were unable to read and write, one of their guardians (including mother, father, grandparents, and babysitters) was eligible for participating in the study on behalf of the children.

### Sampling and survey procedures

**Fig. 1:**
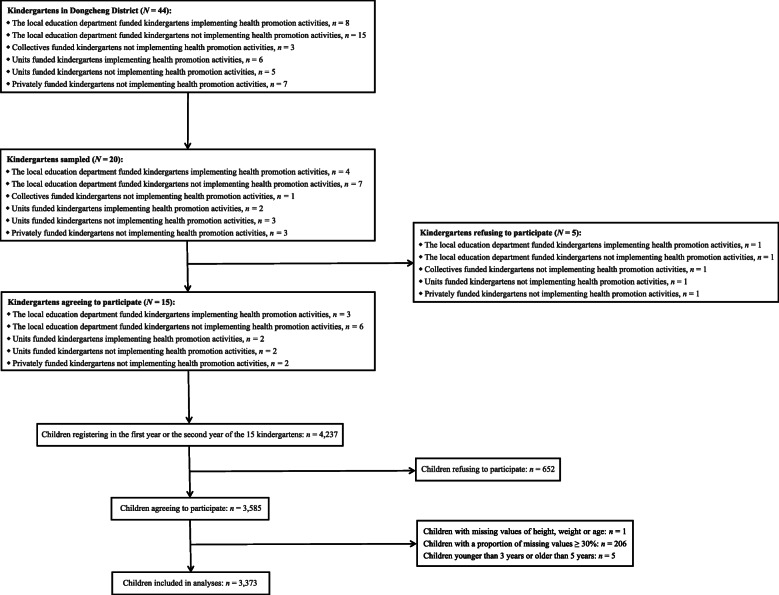
Sampling and Survey Procedures of the Study

Taking each class as a cluster, a stratified proportionate cluster sampling was used. The number of classes to be recruited was determined by a sample size estimation formula: $N=k\times \frac{u_{\alpha}^2P\left(1-P\right)}{\delta^2}$. Assuming the prevalence of overweight/obesity among children in the first and second years of kindergarten in the Dongcheng District (*P*) was 15% (estimated through a pilot study), a two-sided significance level of 5% (*u*_*α*_ = 1.96), a minimally detectable rate difference (*δ*) of 1.5%, and a 10% non-response rate and a 1.5 design effect of cluster sampling (*k* = 1.65), the number of child-guardian dyads needed was calculated to be 3592. Assume that there are 20 to 30 children per class and 4 to 10 classes of the first and second years per kindergarten, approximately 150 classes of 20 kindergartens were required. A full list of the forty-four kindergartens in the Dongcheng District was extracted from the local education bureau, and stratified by financing sources and implementation of health promotion activities. Random numbers were generated to select kindergartens by stratification. Heads of the selected twenty kindergartens were contacted before the survey for their approval of participation, and fifteen agreed. With written informed consent from guardians, all of the 4237 children in classes of the first and second years from the fifteen kindergartens, along with one of their guardians, were invited to participate in the survey from April 2018 till the end of the Spring Semester of 2017/2018 Academic Year. To obtain dietary consumption frequencies and essential covariates, questionnaires with unique ID numbers were distributed by teachers who were in charge of classes. All teachers in this study received standardized training and were responsible for providing necessary instructions to guardians. Guardians then took questionnaires home and completed them anonymously on behalf of their children, considering many preschool children were not capable of reading and writing. One week later, questionnaires were collected and preliminarily checked by teachers in charge of classes. Blank questionnaires were permitted if guardians refused to participate. A total of 3585 child-guardian dyads participated in the survey, with a response rate of 84.61% (3585/4237). We excluded 212 dyads due to incomplete information. Figure 1 provides details on sampling procedures.

### Measurements

#### Weight and height

Children’s weight and height were measured annually (usually at the end of a semester, which is close to the survey date) by trained school nurses following standardized anthropometry measurement protocols developed by the Child Care Center of Bejing Health Bureau to the nearest 0.1 kg and 0.1 cm, with children wearing light clothes and no shoes. Each child’s weight and height were also attached to a unique ID number, enabling matching with the questionnaire data. Children’s body mass index (BMI) was calculated, and weight status was defined by criteria developed by the World Health Organization (WHO) [24–26]: the children aged three to four whose BMI Z-scores greater than 2 standard deviations (*SD*s) and 3 *SD*s from the median of their age and gender group were classified as overweight and obesity, respectively, and for children aged five, the cut-points were 1 *SD* and 2 *SD*s.

#### Dietary consumption frequencies

According to *The Dietary Guidelines for Chinese Preschool Children 2016* [27], a validated food frequency questionnaire (FFQ) [28] was adapted and developed specifically for the study to measure children’s usual consumption frequencies of twenty-five food and beverage groups frequently consumed by the Chinese with seven levels (never, less than once a week, once a week, twice to four times a week, five to six times a week, seven times a week, and more than seven times a week) scored 0 to 6, and several examples were annotated for some confusing groups to improve interpretability. Portion sizes were not collected, as better validity correlations were obtained in FFQs which did not assess them [29]. Additional file 1 is an English version of the FFQ. Food and beverage groups were deemed to be “consumed” when their consumption frequencies were between less than once a week and more than seven times a week. Test-retest reliability of the FFQ was pretested in a pilot study among a convenience sample of 175 pairs of preschool children and guardians, and the results are available in Additional file 2. Given the low consumption frequencies and poor reliability of coffee drinks and energy drinks or sports drinks, they were eliminated from the analyses of dietary patterns and their associations with overweight/obesity. In that condition, the reliability of the adapted FFQ was moderate and comparable to the original one [28].

#### Covariates

Age, gender, the daily average time of moderate-to-vigorous physical activities (MVPA) on weekdays and weekends, and the daily average sedentary time on weekdays and weekends of the children, as well as the highest level of educational attainment, occupation, weight, and the height of their parents were collected via single questions in the questionnaire. Parents’ SES scores [30] and BMI were calculated, and their weight status was defined by criteria developed by the Working Group on Obesity in China (WGOC) [31]: BMI of 24 kg/m^2^ and 28 kg/m^2^ were taken as cut-points for overweight and obesity, respectively.

### Statistical analyses

Continuous variables with normal distribution, continuous variables with skewed distribution, and categorical variables were presented as means (*SD*s), medians (25th and 75th percentiles), and frequencies (percentages), respectively. Dietary patterns were identified through exploratory factor analysis (EFA) [32] whose applicability was confirmed by Bartlett’s test of sphericity and Kaiser-Meyer-Olkin (KMO) value. The EFA was considered to be appropriate if the Bartlett’s test was significant and the KMO value was greater than 0.5 [33]. The number of retained factors was determined by eigenvalues (greater than 1) and interpretability, and factors were rotated with an orthogonal (varimax) rotation to minimize mutual correlations and improve interpretability. Factor loadings represented correlations between each food and beverage group and each factor – loadings greater than 0.3 stood for positive correlations, while those less than − 0.3 for negative. Following the description of these correlations, each factor was named as a specific dietary pattern. Through weighting standardized consumption frequencies of food and beverage groups by their factor loadings and adding up all these values, pattern-specific factor scores were calculated for each child, and the dietary pattern with the largest factor score was defined as the predominant dietary pattern. Differences across predominant dietary patterns in children’s age were tested by one-way analysis of variance, in children’s gender and consumption proportions of food and beverage groups by Pearson’s chi-square tests, and in children’s BMI and weight status, parents’ SES scores and weight status, and consumption frequencies of food and beverage groups by Kruskal-Wallis tests. Considering the multi-stage sampling procedure adopted where classes were taken as clusters, two-level random-intercept logistic models with cluster-robust standard errors, extensions of standard logistic models that treated intercepts as random variables to account for the clustering of one-level units (children) within two-level units (classes) [34], were conducted to estimate the associations between predominant dietary patterns and overweight/obesity, a binary outcome. A null model without any independent variable was run firstly to verify the hierarchy of data, and subsequently, model 1 (only including predominant dietary patterns), model 2 (including adjustment for children’s age and gender), model 3 (including further adjustment for children’s daily average time of MVPA and daily average sedentary time, on weekdays and weekends respectively), and model 4 (including further adjustment for parents’ SES scores and BMI) were fitted. Correlations were expressed as odds ratios (*OR*s) with 95% confidence intervals (*CI*s). Please refer to Additional file 3 for variable definitions and Stata codes of these models. Lastly, a sensitivity analysis was carried out in the same way as model 4, but defining overweight/obesity by criteria specific to Chinese preschool children [35]. Data imputation was applied in the EFA and the two-level models by using means, medians, and modes to replace missing data in continuous variables with normal distribution, continuous variables with skewed distribution, and categorical variables, respectively. All the analyses were completed using Stata/SE 16.0 for Windows (StataCorp, College Station, Texas, USA). Statistical significance was considered when *P* ≤ 0.05 (two-sided).

## Results

Table 1 provides the characteristics of the children and their parents. A total of 3373 children with a mean age of 4.24 (*SD*: 0.67) years participated in the study. Over half (52.33%) of them were boys, and 8.19% were either overweight or obese. The majority (94.40%) of the guardians who participated in the survey were either children’s mothers (71.01%) or fathers (23.39%). About one fifth (17.79%) of the mothers and over half (57.58%) of the fathers were classified as either overweight or obese.

**Table 1:** Characteristics of Participants (*N =* 3373)

Characteristics	Statistics
Children	
Children’s age (years), mean (*SD*)	4.24 (0.67)
Three-year-old, *n* (%)	464 (13.76)
Four-year-old, *n* (%)	1652 (48.98)
Five-year-old, *n* (%)	1257 (37.27)
Children’s gender, *n* (%)	
Boy	1765 (52.33)
Girl	1608 (47.67)
Children’s grades, *n* (%)	
The first year of kindergarten	1736 (51.47)
The second year of kindergarten	1637 (48.53)
Children’s BMI (kg/m^2^), *M* (*Q1*, *Q2*)	15.34 (14.49, 16.27)
Children’s weight status, *n* (%)	
Underweight or normal weight	3097 (91.82)
Overweight	179 (5.31)
Obesity	97 (2.88)
Relationships between participating guardians and children, *n* (%)	
Father-child	789 (23.39)
Mother-child	2395 (71.01)
Other relationships	160 (4.74)
Data missing	29 (0.86)
Children’s parents	
Parents’ age^a^ (years), mean (*SD*)	
Father	37.12 (4.39)
Mother	35.16 (3.45)
Parents’ highest level of educational attainment, *n (%)*	
Father	
Technical college or below	778 (23.07)
University	1600 (47.44)
Postgraduate or above	956 (28.34)
Data missing	39 (1.16)
Mother	
Technical college or below	657 (19.48)
University	1789 (53.04)
Postgraduate or above	882 (26.15)
Data missing	45 (1.33)
Parents’ occupations, *n (%)*	
Father	
Administrative	785 (23.27)
Technician	839 (24.87)
Clerk	1048 (31.07)
Other occupation	623 (18.47)
Data missing	78 (2.31)
Mother	
Administrative	652 (19.33)
Technician	746 (22.12)
Clerk	1245 (36.91)
Other occupation	639 (18.94)
Data missing	91 (2.70)
Parents’ weight status, *n* (%)	
Father	
Underweight or normal weight	1276 (37.83)
Overweight	1394 (41.33)
Obesity	548 (16.25)
Data missing	155 (4.60)
Mother	
Underweight or normal weight	2646 (78.45)
Overweight	507 (15.03)
Obesity	93 (2.76)
Data missing	127 (3.77)

Abbreviations: *SD*: standard deviation; BMI: body mass index; *M* (*Q1*, *Q2*): median (25th and 75th percentiles)

Note: ^a^Missing data exists

Table 2 shows consumption proportions and frequencies for each food and beverage group. Nearly two-thirds of the children consumed fruits (71.66%) and vegetables (61.52%) more than seven times a week, while the proportion was smaller for milk (49.81%) and yogurt or other dairy products (30.74%). Meanwhile, approximately one fifth (21.20%) of the children did not consume any one of the seven SSB groups (flavored milk drinks, carbonated drinks, flavored fruit/vegetable drinks, energy drinks or sports drinks, tea drinks, plant-protein drinks, and coffee drinks), and only 11 children consumed none of the five high-energy snack groups (sweets, pastries, puffed foods, fried foods, and western fast foods).

**Table 2:** Consumption Proportions and Frequencies of Food and Beverage Groups (*N =* 3373)

Food and beverage groups	Consumption proportions with 95% *CI*s (%)	Consumption frequencies, *n* (%)						Data missing
		Less than once a week	Once a week	Twice to four times a week	Five to six times a week	Seven times a week	More than seven times a week	
Fruits	99.91 (99.74, 99.98)	6 (0.18)	13 (0.39)	125 (3.71)	282 (8.36)	491 (14.56)	2417 (71.66)	36 (1.07)
Vegetables	99.85 (99.65, 99.95)	17 (0.50)	49 (1.45)	269 (7.98)	360 (10.67)	520 (15.42)	2075 (61.52)	78 (2.31)
Dark-green vegetables	99.50 (99.19, 99.71)	113 (3.35)	276 (8.18)	1085 (32.17)	687 (20.37)	391 (11.59)	691 (20.49)	113 (3.35)
Other dark-color vegetables	99.70 (99.46, 99.86)	52 (1.54)	230 (6.82)	1000 (29.65)	775 (22.98)	471 (13.96)	771 (22.86)	64 (1.90)
Fresh fruit/vegetable juice	84.73 (83.47, 85.93)	1061 (31.46)	691 (20.49)	695 (20.60)	151 (4.48)	87 (2.58)	116 (3.44)	57 (1.69)
Soybean milk	68.84 (67.25, 70.40)	1166 (34.57)	599 (17.76)	372 (11.03)	59 (1.75)	29 (0.86)	46 (1.36)	51 (1.51)
Milk	97.48 (96.89, 97.98)	119 (3.53)	135 (4.00)	471 (13.96)	389 (11.53)	483 (14.32)	1680 (49.81)	11 (0.33)
Yogurt or other dairy products	98.55 (98.08, 98.92)	112 (3.32)	170 (5.04)	774 (22.95)	620 (18.38)	594 (17.61)	1037 (30.74)	17 (0.50)
Flavored milk drinks	54.52 (52.82, 56.21)	944 (27.99)	320 (9.49)	299 (8.86)	88 (2.61)	58 (1.72)	66 (1.96)	64 (1.90)
Carbonated drinks	26.50 (25.02, 28.03)	608 (18.03)	142 (4.21)	79 (2.34)	22 (0.65)	5 (0.15)	10 (0.30)	28 (0.83)
Flavored fruit/vegetable drinks	53.16 (51.46, 54.85)	1172 (34.75)	343 (10.17)	196 (5.81)	26 (0.77)	19 (0.56)	17 (0.50)	20 (0.59)
Energy drinks or sports drinks	16.34 (15.10, 17.63)	389 (11.53)	84 (2.49)	34 (1.01)	10 (0.30)	4 (0.12)	4 (0.12)	26 (0.77)
Tea drinks	20.49 (19.14, 21.89)	453 (13.43)	112 (3.32)	58 (1.72)	7 (0.21)	5 (0.15)	11 (0.33)	45 (1.33)
Plant-protein drinks	44.41 (42.73, 46.11)	1081 (32.05)	246 (7.29)	97 (2.88)	24 (0.71)	9 (0.27)	11 (0.33)	30 (0.89)
Coffee drinks	3.11 (2.55, 3.76)	53 (1.57)	5 (0.15)	4 (0.12)	7 (0.21)	2 (0.06)	3 (0.09)	31 (0.92)
Sweets	96.74 (96.08, 97.31)	673 (19.95)	704 (20.87)	1244 (36.88)	338 (10.02)	142 (4.21)	145 (4.30)	17 (0.50)
Pastries	97.51 (96.93, 98.01)	757 (22.44)	939 (27.84)	1188 (35.22)	251 (7.44)	77 (2.28)	49 (1.45)	28 (0.83)
Puffed foods	71.51 (69.95, 73.03)	1491 (44.20)	552 (16.37)	291 (8.63)	25 (0.74)	13 (0.39)	6 (0.18)	34 (1.01)
Fried foods	79.63 (78.23, 80.98)	1729 (51.26)	667 (19.77)	227 (6.73)	19 (0.56)	8 (0.24)	4 (0.12)	32 (0.95)
Western fast foods	83.04 (81.73, 84.29)	1951 (57.84)	602 (17.85)	175 (5.19)	19 (0.56)	10 (0.30)	8 (0.24)	36 (1.07)
Nuts	95.14 (94.36, 95.84)	721 (21.38)	819 (24.28)	1086 (32.20)	325 (9.64)	110 (3.26)	119 (3.53)	29 (0.86)
Wheat or wheat foods	86.87 (85.68, 87.99)	1052 (31.19)	898 (26.62)	667 (19.77)	165 (4.89)	64 (1.90)	50 (1.48)	34 (1.01)
Meat or poultry	99.58 (99.30, 99.77)	59 (1.75)	120 (3.56)	676 (20.04)	714 (21.17)	619 (18.35)	1142 (33.86)	29 (0.86)
Fishery products	98.49 (98.02, 98.87)	271 (8.03)	715 (21.20)	1417 (42.01)	486 (14.41)	205 (6.08)	196 (5.81)	32 (0.95)
Other protein-rich foods	99.58 (99.30, 99.77)	48 (1.42)	181 (5.37)	822 (24.37)	704 (20.87)	661 (19.60)	921 (27.31)	22 (0.65)

Abbreviation: *CI*: confidence interval.

The Bartlett’s test of sphericity was significant (*P* <  0.001) and the KMO value was 0.828, which suggests that EFA is applicable. Although there were six factors with eigenvalues greater than 1, only four were retained by the reason of interpretability, which altogether accounted for 43.22% of the total variance. Table 3 presents loadings on the four factors of food and beverage groups after rotation. The first factor was named the “SSB and snack” pattern, as being positively related to fresh fruit/vegetable juice, flavored milk drinks, carbonated drinks, flavored fruit/vegetable drinks, tea drinks, plant-protein drinks, puffed foods, fried foods, and Western fast foods. Likewise, the second factor characterized by fruits, vegetables, dark-green vegetables, other dark-color vegetables, meat or poultry, and other protein-rich foods was named the “Chinese traditional” pattern. The third pattern characterized by soybean milk, milk, yogurt or other dairy products, nuts, wheat or wheat foods, fishery products, and other protein-rich foods was named the “Health conscious” pattern. The fourth pattern characterized by sweets, pastries, puffed foods, fried foods, and Western fast foods was named the “Snack” pattern. Among the children, 21.02% (95% *CI*: 19.68 to 22.43%) were predominated by the “SSB and snack” pattern, 27.78% (95% *CI*: 26.29 to 29.32%) by the “Chinese traditional” pattern, 24.90% (95% *CI*: 23.47 to 26.39%) by the “Health conscious” pattern, and 26.30% (95% *CI*: 24.84 to 27.81%) by the “Snack” pattern.

**Table 3:** Factor Loadings after Orthogonal Rotation on Idenfied Dietary Patterns of Food and Beverage Groups^a, b^ (*N =* 3373)

Food and beverage groups^c^	“Sugar-sweetened beverage and snack” pattern	“Chinese traditional” pattern	“Health conscious” pattern	“Snack” pattern
Fruits		0.67		
Vegetables		0.77		
Dark-green vegetables		0.68		
Other dark-color vegetables		0.66		
Fresh fruit/vegetable juice	0.39		0.45	
Soybean milk	0.48		0.44	
Milk			0.42	
Yogurt or other dairy products			0.36	
Flavored milk drinks	0.58			
Carbonated drinks	0.63			
Flavored fruit/vegetable drinks	0.66			
Tea drinks	0.64			
Plant-protein drinks	0.63			
Sweets				0.70
Pastries			0.32	0.63
Puffed foods	0.41			0.63
Fried foods	0.43			0.62
Western fast foods	0.37			0.47
Nuts			0.56	
Wheat or wheat foods			0.65	
Meat or poultry		0.46		
Fishery products			0.53	
Other protein-rich foods		0.42	0.42	
Percentages of variation (%)	12.89	11.03	9.70	9.60

Notes: ^a^Blanks represent factor loadings with absolute values less than 0.3; ^b^Data imputation was applied by using modes to replace missing data for each food and beverage group; ^c^Coffee drinks and energy drinks or sports drinks were eliminated

Table 4 describes the differences in participants’ characteristics and consumption proportions of food and beverage groups by predominant dietary patterns, and Additional file 4 includes more detailed information on the differences in consumption frequencies of food and beverage groups. In relative to other dietary patterns, BMI medians of the children predominated by the “SSB and snack” pattern and the “Snack” pattern were larger, but parents’ SES scores were smaller for the children predominated by the “SSB and snack” pattern. Apart from fruits, vegetables, and yogurt or other dairy products, consumption proportions of 20 food and beverage groups were statistically different across predominant dietary patterns.

**Table 4:** Differences in Participants’ Characteristics and Consummption Proportions of Food and Beverage Groups by Predominant Dietary Patterns (*N =* 3373)

Variables	“Sugar-sweetened beverage and snack” pattern	“Chinese traditional” pattern	“Health conscious” pattern	“Snack” pattern	*F* values/Chi-square values^a^	*P* values
Participants’ characteristics						
Children’s age (years), mean (*SD*)	4.34 (0.64)	4.21 (0.68)	4.18 (0.66)	4.23 (0.69)	8.894	< 0.001
Children’s gender, *n* (*%*)					7.727	0.052
Boy	377 (53.17)	504 (53.79)	455 (54.17)	429 (48.37)		
Girl	332 (46.83)	433 (46.21)	385 (45.83)	458 (51.63)		
Children’s BMI (kg/m^2^), *M* (*Q1*, *Q2*)	15.44 (14.54, 16.47)	15.26 (14.48, 16.22)	15.23 (14.43, 16.12)	15.42 (14.56, 16.29)	13.156	0.004
Children’s weight status, *n* (%)					17.068	< 0.001
Underweight or normal-weight	625 (88.15)	873 (93.17)	783 (93.21)	816 (92.00)		
Overweight	54 (7.62)	39 (4.16)	35 (4.17)	51 (5.75)		
Obesity	30 (4.23)	25 (2.67)	22 (2.62)	20 (2.25)		
Parents’ SES scores^b^, *M* (*Q1*, *Q2*)						
Father	63.7 (59.3, 69.3)	67.7 (62.5, 69.5)	67.7 (62.5, 69.3)	66.7 (62.1, 69.3)	44.921	< 0.001
Mother	64.6 (61.4, 69.8)	69.5 (64.6, 71.4)	69.5 (64.6, 71.4)	69.5 (64.6, 71.4)	53.407	< 0.001
Parents’ weight status, *n* (%)						
Father					5.407	0.144
Underweight or normal weight	259 (36.53)	368 (39.27)	322 (38.33)	327 (36.87)		
Overweight	287 (40.48)	384 (40.98)	376 (44.76)	347 (39.12)		
Obesity	122 (17.21)	147 (15.69)	107 (12.74)	172 (19.39)		
Data missing	41 (5.78)	38 (4.06)	35 (4.17)	41 (4.62)		
Mother					14.862	0.002
Underweight or normal weight	527 (74.33)	732 (78.12)	695 (82.74)	692 (78.02)		
Overweight	122 (17.21)	149 (15.90)	104 (12.38)	132 (14.88)		
Obesity	29 (4.09)	21 (2.24)	16 (1.90)	27 (3.04)		
Data missing	31 (4.37)	35 (3.74)	25 (2.98)	36 (4.06)		
Consumption proportions of food and beverage groups^b^, *n* (*%*)						
Fruits	708 (99.86)	937 (100.00)	839 (99.88)	886 (99.89)	1.195	0.750
Vegetables	709 (100.00)	937 (100.00)	838 (99.76)	884 (99.66)	5.067	0.170
Dark-green vegetables	706 (99.58)	937 (100.00)	834 (99.29)	879 (99.10)	8.542	0.036
Other dark-color vegetables	709 (100.00)	937 (100.00)	840 (100.00)	877 (98.87)	27.988	< 0.001
Fresh fruit/vegetable juice	646 (91.11)	751 (80.15)	764 (90.95)	697 (78.58)	88.354	< 0.001
Soybean milk	586 (82.65)	610 (65.10)	668 (79.52)	458 (51.63)	232.325	< 0.001
Milk	687 (96.90)	911 (97.23)	835 (99.40)	855 (96.39)	18.216	< 0.001
Yogurt or other dairy products	694 (97.88)	921 (98.29)	835 (99.40)	874 (98.53)	6.878	0.076
Flavored milk drinks	579 (81.66)	398 (42.48)	367 (43.69)	495 (55.81)	313.913	< 0.001
Carbonated drinks	404 (56.98)	173 (18.46)	95 (11.31)	222 (25.03)	488.557	< 0.001
Flavored fruit/vegetable drinks	565 (79.69)	390 (41.62)	315 (37.50)	523 (58.96)	347.720	< 0.001
Tea drinks	344 (48.52)	113 (12.06)	68 (8.10)	166 (18.71)	478.026	< 0.001
Plant-protein drinks	513 (72.36)	279 (29.78)	365 (43.45)	341 (38.44)	317.776	< 0.001
Sweets	686 (96.76)	886 (94.56)	808 (96.19)	883 (99.55)	37.072	< 0.001
Pastries	680 (95.91)	898 (95.84)	828 (98.57)	883 (99.55)	37.337	< 0.001
Puffed foods	596 (84.06)	563 (60.09)	474 (56.43)	779 (87.82)	327.716	< 0.001
Fried foods	631 (89.00)	667 (71.18)	566 (67.38)	822 (92.67)	248.043	< 0.001
Western fast foods	622 (87.73)	719 (76.73)	660 (78.57)	800 (90.19)	82.033	< 0.001
Nuts	668 (94.22)	874 (93.28)	830 (98.81)	837 (94.36)	34.076	< 0.001
Wheat or wheat foods	609 (85.90)	770 (82.18)	821 (97.74)	730 (82.30)	121.404	< 0.001
Meat or poultry	700 (98.73)	936 (99.89)	837 (99.64)	886 (99.89)	16.662	< 0.001
Fishery products	686 (96.76)	927 (98.93)	833 (99.17)	876 (98.76)	18.684	< 0.001
Other protein-rich foods	703 (99.15)	936 (99.89)	840 (100.00)	880 (99.21)	11.891	0.008

Abbreviations: *SD*: standard deviation; BMI: body mass index; *M* (*Q1*, *Q2*): median (25th and 75th percentiles); SES: socioeconomic status

Notes: ^a^Chi-square values of the Kruskal-Wallis tests for children’s body mass index and weight status, and parents’ socioeconomic status scores were adjusted for ties; ^b^Missing data exists

The null model confirmed the hierarchy of data (intraclass correlation coefficient = 0.10, 95% *CI*: 0.06 to 0.18). Table 5 displays the associations between predominant dietary patterns and overweight/obesity. After adjusting for potential confounders, the “SSB and snack” pattern was positively related to overweight/obesity — compared with the “Chinese traditional” pattern, the odds of being overweight/obesity for the children predominated by the “SSB and snack” pattern increased to 1.61 (95% *CI*:1.09 to 2.38).

**Table 5:** Associations between Predominant Dietary Patterns and Overweight/Obesity^a, b, c^ (*N =* 3373)

Predominant dietary patterns^d^	*OR*s with 95% *CI*s/Variance with 95% *CI*s	Robust *SE*s	*Z* values	*P* values
Fixed parts				
“Sugar-sweetened beverage and snack” pattern				
Model 1	1.76 (1.21, 2.58)	0.34	2.934	0.003
Model 2	1.67 (1.15, 2.43)	0.32	2.689	0.007
Model 3	1.66 (1.14, 2.42)	0.32	2.662	0.008
Model 4	1.61 (1.09, 2.38)	0.32	2.385	0.017
“Health conscious” pattern				
Model 1	1.00 (0.70, 1.43)	0.18	−0.014	0.988
Model 2	1.04 (0.72, 1.51)	0.20	0.226	0.821
Model 3	1.05 (0.73, 1.51)	0.20	0.247	0.805
Model 4	1.13 (0.78, 1.65)	0.22	0.646	0.518
“Snack” pattern				
Model 1	1.16 (0.82, 1.63)	0.20	0.838	0.402
Model 2	1.22 (0.86, 1.72)	0.22	1.102	0.271
Model 3	1.17 (0.83, 1.65)	0.20	0.921	0.357
Model 4	1.13 (0.79, 1.61)	0.20	0.680	0.497
Random parts				
Intercepts at class level				
Model 1	0.35 (0.18, 0.67)	0.12		
Model 2	0.00 (0.00, 0.00)	0.00		
Model 3	0.00 (0.00, 0.00)	0.00		
Model 4	0.02 (0.00, 487.47)	0.08		

Abbreviations: *OR*: odds ratio; *CI*: confidence interval; *SE*: standard error

Notes: ^a^Dependent variable: overweight/obesity; ^b^Model 1 only included predominant dietary patterns; model 2 included adjustment for children’s age and gender; model 3 included further adjustment for children’s daily average time of moderate-to-vigorous physical activities and daily average sedentary time, on weekdays and weekends respectively; model 4 included further adjustment for parents’ socioeconomic status scores and body mass index; ^c^Data imputation was applied by using modes to replace missing data in children’s daily average time of moderate-to-vigorous physical activities and daily average sedentary time, and medians in parents’ socioeconomic status scores and body mass index; ^d^Reference: the “Chinese traditional” pattern

Similar results were obtained from the sensitivity analysis: under a criteria specific to Chinese preschool children, 17.76% (95% *CI*: 16.48 to 19.09%) of the children were either overweight or obese, and compared with the “Chinese traditional” pattern, solely the “SSB and snack” pattern was positively associated with overweight/obesity (*OR*: 1.47, 95% *CI*: 1.12 to 1.91) after controlling for potential confounders.

## Discussion

In the cross-sectional study conducted among preschool children in the Dongcheng District of Beijing, four dietary patterns, i.e., a “SSB and snack” pattern, a “Chinese traditional” pattern, a “Health conscious” pattern, and a “Snack” pattern, were identified. Roughly half of the children had a preference for the “SSB and snack” pattern and the “Snack” pattern which have high energy density but low nutritional value. After controlling for potential confounders, the “SSB and snack” pattern was associated with a higher risk of overweight/obesity, compared with the “Chinese traditional” pattern.

Dietary patterns identified across studies are not the same. This discrepancy could be explained by characteristics of studies, such as the time being carried out, assessment tools adopted, identification processes, and the influences of macro-environments like media/society, food supply, and nutrition-related policies [36]. However, the numbers of dietary patterns identified in previous studies along with their composition and ability to capture overall variance remain relatively stable [36]. As for preschool children, some common dietary patterns considered healthy, less healthy, and traditional have been identified in previous studies [16–21, 23]. They share many characteristics with the dietary patterns identified in the current study. For example, five dietary patterns were identified among children aged three to six in Ma’anshan City, China, including a “Beverage” pattern characterized by flavored milk, drinks, carbonated beverages, and yogurt, a “Protein” pattern characterized by red meat, poultry, egg, fish and other fishery products, and fruits, and a “Snack” pattern characterized by sweets, chocolate, biscuits or cake, puffed foods, and milk-based puddings and custard [18]. Given that the unhealthy eating habits in the early years could track to mid-childhood and even later life [10, 11], it is important to promote healthy dietary patterns among preschool children. Nonetheless, nearly half of the children in the present study were predominated by the “SSB and snack” pattern and the “Snack” pattern, both characterized by diets high in energy density but low in nutritional value. It would be well worth noting that the “Health conscious” pattern characterized by soybean milk, milk, yogurt or other dairy products, nuts, wheat or wheat foods, fishery products, and other protein-rich foods was identified in the present study, as well as a previous study in Wuhu City, China [17], reflecting a possibly raising awareness of nutrition and health among guardians of Chinese preschool children. We expect the “Health conscious” pattern to be a predictor of children’s adherence to healthy dietary patterns, and additional in-depth study to explore the correlates and effects of this pattern would be worthwhile.

In China, preschool children in underdeveloped areas, such as western rural areas, still suffer from undernutrition, while in metropolises, such as Beijing, overweight and obesity are increasingly prevalent [37]. The epidemiological evidence of the associations between dietary patterns and overweight/obesity is inconsistent among the Chinese population — a study among school-aged children and adolescents from seven provinces reported a dietary pattern characterized by fried foods, snacks, western fast foods, soft drinks, and eating outside was a risk factor for overweight/obesity [38], whilst another study conducted among the same age group in Ningxia, an underdeveloped area, did not find such association [39]. One potential explanation of the contradiction in cross-sectional studies is that there might be methodological quality problems. For example, overweight/obese children might have changed their dietary behaviors by the time when the survey was carried out [40]. In the present study, compared with the “Chinese traditional” pattern, the correlation between the “SSB and snack” pattern and overweight/obesity was statistically significant. The identification of this pattern indicates that SSB consumers were more likely to consume snacks as well. According to the hypothesis proposed by a previous study, the preference for sweetness and higher consumption of sweet foods could be caused by repeated exposure to SSBs (or unhealthy snacks) even in a very short period [41]. Therefore, comprehensive measures to simultaneously reduce the exposure to SSBs and unhealthy snacks among children are reasonable and urgent.

In the present study, the children with parents in lower SES had higher risk of being predominated by energy-dense and low-nutrient dietary patterns, which is consistent with previous studies [16, 42, 43]. Existing evidence suggests that parenting practices, such as serving unhealthy foods and beverages at meals and providing them to children whenever they want, mediate the association between parents’ SES and children’s unhealthy eating [44, 45]. Therefore, it is crucial to master correct parenting practices, especially for low SES parents. Well-educated (a characteristic of high SES) parents may have better knowledge and ability needed for understanding and using nutrition labels [46, 47], and are more likely to choose healthy foods for their children [48, 49]. Thus, it is beneficial to help low SES parents improve knowledge and ability for understanding and using nutrition labels. Besides, imposing taxes on SSBs and unhealthy snacks to control demands for them has also been proved effective among low SES population in some countries [50, 51], as when the cost of these products get higher, the availability of these products might accordingly become lower in low SES families [52]. Further studies should be conducted in China to help children move towards a healthier diet.

The findings of the present study should be interpreted with consideration of the following limitations. Firstly, the study was based on a cross-sectional survey, where children’s dietary consumption and weight status were obtained at the same time, leading to difficulties in establishing temporality relationships. Secondly, among the twenty representative kindergartens initially sampled, five declined to participate, which may weaken the generalizability of the results. Thirdly, this study got responses from the children’s guardians, and they might be unable to accurately capture children’s dietary consumption and physical activities out of home (mainly at kindergartens). This limitation may not have major impacts on the results, since guardians were in close contact with kindergartens to keep track of their children’s dietary, physical activities, etc. Fourthly, the EFA adopted to identify dietary patterns involved several arbitrary decisions, including the number of factors to extract and the method of rotation [53]. Hence, dietary patterns identified in the present study might be difficult to exactly replicate in other populations. Finally, the associations between predominant dietary patterns and overweight/obesity were possibly biased by residual confounding, especially from variables difficult to measure precisely by the questionnaire, such as dietary consumption and physical activities. This problem could be dealt with in future studies by adopting more objective and accurate measurement tools, such as wearable automated cameras and accelerometers.

## Conclusions

Nearly half of the children were predominated by dietary patterns with high energy density but low nutritional value. Besides, compared with the “Chinese traditional” pattern, the “SSB and snack” pattern (high in SSB and snack consumption) was positively associated with overweight/obesity. To address the childhood obesity problem in China, particularly in metropolises, these findings highlight the importance and urgency of taking comprehensive measures to simultaneously reduce consumption of SSBs and unhealthy snacks among preschool children.

## Supplementary Information

**Additional file 1.** An English Version of the Food Frequency Questionnaire. An English version of the FFQ adopted in the study.

**Additional file 2.** Test-retest Reliability of the Food Frequency Questionnaire. Methods and results of test-retest reliability evaluation on the FFQ adopted in the study.

**Additional file 3.** Variable Definitions of Two-level Random-intercept Logistic Models & Stata Codes of Two-level Random-intercept Logistic Models. Variables and their definitions, as well as Stata codes of the two-level random-intercept logistic models to test the associations between predominant dietary patterns and overweight/obesity.

**Additional file 4.** Differences in Consumption Frequencies of Food and Beverage Groups by Predominant Dietary Patterns. Detailed results of differences in consumption frequencies of food and beverage groups by predominant dietary patterns.

## Data Availability

According to private and confidential clauses stated in the informed consent, the dataset generated and analysed during the current study is ethically restricted and not publicly available. It would be available from Prof. Juan ZHANG (E-mail: zhangjuan@sph.pumc.edu.cn) on reasonable request.

## Abbreviations

BMI: body mass index
*CI*: confidence interval
EFA: exploratory factor analysis
FFQ: food frequency questionnaire
KMO value: Kaiser-Meyer-Olkin value
*M* (*Q1*, *Q2*): median (25th and 75th percentiles)
MVPA: moderate-to-vigorous physical activities
*OR*: odds ratio
*SD*: standard deviation
*SE*: standard error
SES: socioeconomic status
SSB: sugar-sweetened beverage
WGOC: Working Group on Obesity in China
WHO: World Health Organization
