# Supplementary material for: Dietary patterns and their associations with overweight/obesity among preschool children in Dongcheng District of Beijing: a cross-sectional study
Source: BMC Public Health. 2021 Jan 27;21:223. doi: 10.1186/s12889-021-10240-x (PMC7839210; doi:10.1186/s12889-021-10240-x)
Supplement: Supplementary file 1 — Additional file 1. An English Version of the Food Frequency Questionnaire. An English version of the FFQ adopted in the study. [file 12889_2021_10240_MOESM1_ESM.docx]

**An English Version of the Food Frequency Questionnaire**

How many times does your child usually eat . . . ? (Please mark only one box for each line)

| Food and beverage groups | Never | Less than once a week | Once a week | Twice to four times a week | Five to six times a week | Seven times a week | More than seven times a week |
| --- | --- | --- | --- | --- | --- | --- | --- |
| Fruits |  |  |  |  |  |  |  |
| Vegetables |  |  |  |  |  |  |  |
| Dark-green vegetables (broccoli, spinach, etc.) |  |  |  |  |  |  |  |
| Other dark-color vegetables (tomato, carrot, pumpkin, amaranth, purple cabbage, etc.) |  |  |  |  |  |  |  |
| Fresh fruit/vegetable juice |  |  |  |  |  |  |  |
| Soybean milk |  |  |  |  |  |  |  |
| Milk (pure milk, skimmed milk, milk powder, etc.) |  |  |  |  |  |  |  |
| Yogurt or other dairy products (milk slices, condensed milk, cheese, etc.) |  |  |  |  |  |  |  |
| Flavored milk drinks (Peanut Milk, milk tea, etc.) |  |  |  |  |  |  |  |
| Carbonated drinks (Coke, Sprite, etc.) |  |  |  |  |  |  |  |
| Flavored fruit/vegetable drinks (Orange Multi, Minute Maid, etc.) |  |  |  |  |  |  |  |
| Energy drinks or sports drinks (Red Bull, Pulsation, etc.) |  |  |  |  |  |  |  |
| Tea drinks (Iced Black Tea, Jasmine Tea, etc.) |  |  |  |  |  |  |  |
| Plant-protein drinks (walnut syrup, almond syrup, etc.) |  |  |  |  |  |  |  |
| Coffee drinks (Nescafe, Fire Coffee, etc.) |  |  |  |  |  |  |  |
| Sweets (candies, ice cream, chocolate, etc.) |  |  |  |  |  |  |  |
| Pastries (cake, Chinese cake, donuts, etc.) |  |  |  |  |  |  |  |
| Puffed foods (potato chips, shrimp chips, etc.) |  |  |  |  |  |  |  |
| Fried foods (French fries, fried chicken, etc.) |  |  |  |  |  |  |  |
| Western fast foods (hamburgers, pizza, etc.) |  |  |  |  |  |  |  |
| Nuts (melon seeds, peanuts, walnuts, etc.) |  |  |  |  |  |  |  |
| Wheat or wheat foods (whole wheat bread, cereal, etc.) |  |  |  |  |  |  |  |
| Meat or poultry (pork, chicken, etc.) |  |  |  |  |  |  |  |
| Fishery products (fish, shrimp, crab, etc.) |  |  |  |  |  |  |  |
| Other protein-rich foods (beans or bean products, eggs, etc.) |  |  |  |  |  |  |  |
