# Supplementary material for: Dietary patterns and their associations with overweight/obesity among preschool children in Dongcheng District of Beijing: a cross-sectional study
Source: BMC Public Health. 2021 Jan 27;21:223. doi: 10.1186/s12889-021-10240-x (PMC7839210; doi:10.1186/s12889-021-10240-x)
Supplement: Supplementary file 2 — Additional file 2. Test-retest Reliability of the Food Frequency Questionnaire. Methods and results of test-retest reliability evaluation on the FFQ adopted in the study. [file 12889_2021_10240_MOESM2_ESM.docx]

**Test-retest Reliability of the Food Frequency Questionnaire**

The food frequency questionnaire adopted in the study was adapted from the one in the International Study of Childhood Obesity, Lifestyle and the Environment, whose reliability and validity had already been tested (1).To appraise test-retest reliability of the food frequency questionnaire adopted in the study, from two conveniently selected kindergartens in the Dongcheng District of Beijing, 206 pairs of preschool children and their guardians were invited to participate in a pilot study in April 2018. Questionnaires (FFQ-1) were completed by guardians as proxies of their children, with informed consent obtained from them. Two weeks later, the same participants administered the same questionnaire (FFQ-2) repeatedly. A total of 175 guardians completed FFQ-1 and FFQ-2. Wilcoxon signed-rank tests, weighted *Kappa*, and Kendall's coefficients of concordance were used to evaluate the agreement degree between FFQ-1 and FFQ-2.

Table 1 presents results of test-retest reliability, which was moderate and comparable to the original one (1). The measurements of soybean milk were statistically different between FFQ-1 and FFQ-2. Values of weighted *Kappa* ranged from -0.01 (coffee drinks) to 0.60 (milk and carbonated drinks). All of them were statistically significant but coffee drinks. Kendall's coefficients of concordance ranged from 0.03 (coffee drinks) to 0.71 (fresh fruit/vegetable juice). Considering low consumption frequencies and poor reliability of coffee drinks and energy drinks or sports drinks, we eliminated them from data analyses.

**Table 1. Test-retest Reliability of the Food Frequency Questionnaire^a^ (*N* = 175)**

| Food and beverage groups | FFQ-1, *M* (*Q_1_*, *Q_2_*) | FFQ-2, *M* (*Q_1_*, *Q_2_*) | Wilcoxon signed-rank exact *P* values | Weighted *Kappa* values | Kendall's coefficients of concordance |
| --- | --- | --- | --- | --- | --- |
| Fruits | 6 (6, 6) | 6 (5, 6) | 0.119 | 0.30^***^ | 0.32 |
| Vegetables | 6 (5, 6) | 6 (5, 6) | 0.107 | 0.38^***^ | 0.48 |
| Dark-green vegetables | 4 (3, 6) | 4 (3, 5) | 0.146 | 0.50^***^ | 0.68^**^ |
| Other dark-color vegetables | 4 (3, 6) | 4 (3, 5) | 0.075 | 0.38^***^ | 0.64^**^ |
| Fresh fruit/vegetable juice | 1 (1, 3) | 2 (1, 3) | 0.948 | 0.59^***^ | 0.71^***^ |
| Soybean milk | 1 (0, 2) | 1 (0, 2) | 0.008 | 0.34^***^ | 0.64^*^ |
| Milk | 6 (4, 6) | 5 (4, 6) | 0.103 | 0.60^***^ | 0.67^**^ |
| Yogurt or other dairy products | 4 (3, 6) | 4 (3, 6) | 0.214 | 0.39^***^ | 0.67^**^ |
| Flavored milk drinks | 1 (0, 1) | 1 (0, 2) | 0.495 | 0.37^***^ | 0.61^*^ |
| Carbonated drinks | 0 (0, 1) | 0 (0, 0) | 0.655 | 0.60^***^ | 0.48 |
| Fruit-flavored drinks or vegetable-flavored drinks | 1 (0, 1) | 1 (0, 1) | 0.697 | 0.35^***^ | 0.62^*^ |
| Energy drinks or sports drinks | 0 (0, 0) | 0 (0, 0) | 0.391 | 0.23^**^ | 0.21 |
| Tea drinks | 0 (0, 0) | 0 (0, 0) | 0.641 | 0.41^***^ | 0.33 |
| Plant-protein drinks | 0 (0, 1) | 0 (0, 1) | 0.797 | 0.52^***^ | 0.61^*^ |
| Coffee drinks | 0 (0, 0) | 0 (0, 0) | 0.622 | -0.01 | 0.03 |
| Sweets | 3 (2, 3) | 3 (2, 3) | 0.501 | 0.55^***^ | 0.69^***^ |
| Pastries | 3 (2, 3) | 3 (2, 3) | 0.999 | 0.42^***^ | 0.64^**^ |
| Puffed foods | 1 (0, 2) | 1 (0, 2) | 0.508 | 0.51^***^ | 0.69^***^ |
| Fried foods | 1 (1, 2) | 1 (1, 2) | 0.721 | 0.51^***^ | 0.66^**^ |
| Western fast foods | 1 (1, 1) | 1 (1, 2) | 0.871 | 0.44^***^ | 0.59^*^ |
| Nuts | 3 (1, 3) | 3 (1, 3) | 0.753 | 0.48^***^ | 0.69^**^ |
| Wheat or wheat foods | 2 (1, 3) | 2 (1, 3) | 0.491 | 0.42^***^ | 0.65^**^ |
| Meat or poultry | 5 (4, 6) | 4 (4, 6) | 0.052 | 0.33^***^ | 0.63^*^ |
| Fishery products | 3 (2, 3) | 3 (2, 4) | 0.166 | 0.41^***^ | 0.64^**^ |
| Other protein-rich foods | 4 (3, 6) | 4 (3, 6) | 0.531 | 0.44^***^ | 0.67^**^ |
| Abbreviations: FFQ-1: the first food frequency questionnaire; *M* (*Q1*, *Q2*): median (25th and 75th percentiles); FFQ-2: the second food frequency questionnaire.  Notes: ^a^Missing data exists; * *P* ≤ 0.05; ** *P* ≤ 0.01; *** *P* ≤ 0.001. | | | | | |

**Reference**

1. Saloheimo T, Gonzalez SA, Erkkola M, et al. The reliability and validity of a short food frequency questionnaire among 9-11-year olds: a multinational study on three middle-income and high-income countries. Int J Obes Suppl. 2015;5(Suppl 2):S22-8. doi:10.1038/ijosup.2015.15
