# Supplementary material for: Dietary patterns and their associations with overweight/obesity among preschool children in Dongcheng District of Beijing: a cross-sectional study
Source: BMC Public Health. 2021 Jan 27;21:223. doi: 10.1186/s12889-021-10240-x (PMC7839210; doi:10.1186/s12889-021-10240-x)
Supplement: Supplementary file 3 — Additional file 3. Variable Definitions of Two-level Random-intercept Logistic Models & Stata Codes of Two-level Random-intercept Logistic Models. Variables and their definitions, as well as Stata codes of the two-level random-intercept logistic models to test the associations between predominant dietary patterns and overweight/obesity. [file 12889_2021_10240_MOESM3_ESM.docx]

# Variable Definitions of Two-level Random-intercept Logistic Models

| Variables | Definitions | Types |
| --- | --- | --- |
| Dependent variable |  |  |
| Overweight/obesity | 1. For children aged three to four, BMI z-scores greater than 2 *SD*s and 3 *SD*s from the age- and gender- specific median were classified as overweight and obesity, respectively (1, 2);  2. For children aged five, BMI z-scores greater than 1 *SD* and 2 *SD*s from the age- and gender- specific median were classified as overweight and obesity, respectively (2, 3).  (BMI is weight in kilograms divided by the square of height in meters.) | A dichotomous variable |
| Independent variables |  |  |
| Predominant dietary pattern | Among four identified dietary patterns, the one with the largest factor score was defined as the predominant dietary pattern. | Three dummy variables |
| Children’s age (years) | — | A continuous variable |
| Children’s gender | — | A dichotomous variable |
| Children’s daily average time of MVPA on weekdays | Guardians’ responses to the question: “In the last Monday to Friday, what is your child(ren)’s daily average time for physical activities that made his/her heartbeat and breathe faster?” Five options of 0 minutes, 1 to 60 minutes, 61 to 120 minutes, 121 to 180 minutes, and more than 180 minutes were scored 0 to 4, respectively. | An ordinal variable |
| Children’s daily average time of MVPA on weekends | Guardians’ responses to the question: “In the last weekends, what is your child(ren)’s daily average time for physical activities that made his/her heartbeat and breathe faster?” Five options of 0 minutes, 1 to 60 minutes, 61 to 120 minutes, 121 to 180 minutes, and more than 180 minutes were scored 0 to 4, respectively. | An ordinal variable |
| Children’s daily average sedentary time on weekdays | Guardians’ responses to the question: “In the last Monday to Friday, what is your child(ren)’s daily average time for sedentary behaviors like sitting still, reading books, watching TV, playing iPads or video games?” Five options of 0 minutes, 1 to 60 minutes, 61 to 120 minutes, 121 to 180 minutes, and more than 180 minutes were scored 0 to 4, respectively. | An ordinal variable |
| Children’s daily average sedentary time on weekends | Guardians’ responses to the question: “In the last weekends, what is your child(ren)’s daily average time for sedentary behaviors like sitting still, reading books, watching TV, playing iPads or video games?” Five options of 0 minutes, 1 to 60 minutes, 61 to 120 minutes, 121 to 180 minutes, and more than 180 minutes were scored 0 to 4, respectively. | An ordinal variable |
| Paternal socioeconomic status score | A score based on the highest level of educational attainment and occupation of each child’s father, reflecting paternal socioeconomic status (4). | A continuous variable |
| Maternal socioeconomic status score | A score based on the highest level of educational attainment and occupation of each child’s mother, reflecting maternal socioeconomic status (4). | A continuous variable |
| Paternal BMI (kg/m^2^) | Father’s weight in kilograms divided by the square of their height in meters. | A continuous variable |
| Maternal BMI (kg/m^2^) | Mother’s weight in kilograms divided by the square of their height in meters. | A continuous variable |
| Abbreviations: BMI: body mass index; *SD*: standard deviation; MVPA: moderate-to-vigorous physical activity. | | |

# Stata Codes of Two-level Random-intercept Logistic Models

**The null model:** melogit *overweight_obesity* || *class*:, vce(cluster *class*) or cformat(%9.2f) pformat(%5.3f) sformat(%8.3f) nolog

**The first model:** melogit *overweight_obesity* ib(2).*pdietpattern* || *class*:, vce(cluster *class*) or cformat(%9.2f) pformat(%5.3f) sformat(%8.3f) nolog

**The second model:** melogit *overweight_obesity* ib(2).*pdietpattern* *c_age* *c_gender* || *class*:, vce(cluster *class*) or cformat(%9.2f) pformat(%5.3f) sformat(%8.3f) nolog

**The third model:** melogit *overweight_obesity* ib(2).*pdietpattern* *c_age* *c_gender* *MVPA_weekdays* *MVPA_weekends* *sedentary_weekdays* *sedentary_weekends* || *class*:, vce(cluster *class*) or cformat(%9.2f) pformat(%5.3f) sformat(%8.3f) nolog

**The fourth model:** melogit *overweight_obesity* ib(2).*pdietpattern* *c_age* *c_gender* *MVPA_weekdays* *MVPA_weekends* *sedentary_weekdays* *sedentary_weekends f_SES m_SES* *f_BMI* *m_BMI* || *class*:, vce(cluster *class*) or cformat(%9.2f) pformat(%5.3f) sformat(%8.3f) nolog

#

**References**

1. World Health Organization. Child growth standards. <http://www.who.int/childgrowth/standards/bmi_for_age/en/>. Accessed 12 October 2018.

2. de Onis M, Lobstein T. Defining obesity risk status in the general childhood population: which cut-offs should we use? Int J Pediatr Obes. 2010;5(6):458-60. doi:10.3109/17477161003615583

3. World Health Organization. Growth reference 5-19 years. <http://www.who.int/growthref/who2007_bmi_for_age/en/>. Accessed 13 October 2018.

4. Green LW. Manual for scoring socioeconomic status for research on health behavior. Public Health Rep. 1970;85(9):815-27. doi:10.2307/4593972
