# Supplementary material for: Dietary patterns and their associations with overweight/obesity among preschool children in Dongcheng District of Beijing: a cross-sectional study
Source: BMC Public Health. 2021 Jan 27;21:223. doi: 10.1186/s12889-021-10240-x (PMC7839210; doi:10.1186/s12889-021-10240-x)
Supplement: Supplementary file 4 — Additional file 4. Differences in Consumption Frequencies of Food and Beverage Groups by Predominant Dietary Patterns. Detailed results of differences in consumption frequencies of food and beverage groups by predominant dietary patterns. [file 12889_2021_10240_MOESM4_ESM.docx]

**Table 1. Differences in Consumption Frequencies of Food and Beverage Groups by Predominant Dietary Patterns (*N =*** **3373)**

| Food and beverage groups^a^ | “Sugar-sweetened beverage and snack” pattern, *n* (*%*) | “Chinese traditional” pattern, *n* (*%*) | “Health conscious” pattern, *n* (*%*) | “Snack” pattern, *n* (*%*) | Chi-square values^b^ | *P* values |
| --- | --- | --- | --- | --- | --- | --- |
| Fruits |  |  |  |  | 354.441 | < 0.001 |
| Never | 1 (0.14) | 0 (0.00) | 1 (0.12) | 1 (0.11) |  |  |
| Less than once a week | 1 (0.14) | 0 (0.00) | 1 (0.12) | 4 (0.45) |  |  |
| Once a week | 7 (0.99) | 0 (0.00) | 4 (0.48) | 2 (0.23) |  |  |
| Twice to four times a week | 59 (8.32) | 0 (0.00) | 21 (2.50) | 45 (5.07) |  |  |
| Five to six times a week | 88 (12.41) | 10 (1.07) | 77 (9.17) | 107 (12.06) |  |  |
| Seven times a week | 137 (19.32) | 39 (4.16) | 150 (17.86) | 165 (18.60) |  |  |
| More than seven times a week | 407 (57.40) | 879 (93.81) | 578 (68.81) | 553 (62.34) |  |  |
| Data missing | 9 (1.27) | 9 (0.96) | 8 (0.95) | 10 (1.13) |  |  |
| Vegetables |  |  |  |  | 467.581 | < 0.001 |
| Never | 0 (0.00) | 0 (0.00) | 2 (0.24) | 3 (0.34) |  |  |
| Less than once a week | 6 (0.85) | 0 (0.00) | 3 (0.36) | 8 (0.90) |  |  |
| Once a week | 12 (1.69) | 0 (0.00) | 18 (2.14) | 19 (2.14) |  |  |
| Twice to four times a week | 75 (10.58) | 2 (0.21) | 78 (9.29) | 114 (12.85) |  |  |
| Five to six times a week | 114 (16.08) | 16 (1.71) | 108 (12.86) | 122 (13.75) |  |  |
| Seven times a week | 154 (21.72) | 58 (6.19) | 154 (18.33) | 154 (17.36) |  |  |
| More than seven times a week | 330 (46.54) | 839 (89.54) | 462 (55.00) | 444 (50.06) |  |  |
| Data missing | 18 (2.54) | 22 (2.35) | 15 (1.79) | 23 (2.59) |  |  |
| Dark-green vegetables |  |  |  |  | 612.954 | < 0.001 |
| Never | 3 (0.42) | 0 (0.00) | 6 (0.71) | 8 (0.90) |  |  |
| Less than once a week | 31 (4.37) | 6 (0.64) | 11 (1.31) | 65 (7.33) |  |  |
| Once a week | 66 (9.31) | 24 (2.56) | 63 (7.50) | 123 (13.87) |  |  |
| Twice to four times a week | 278 (39.21) | 150 (16.01) | 274 (32.62) | 383 (43.18) |  |  |
| Five to six times a week | 141 (19.89) | 187 (19.96) | 195 (23.21) | 164 (18.49) |  |  |
| Seven times a week | 79 (11.14) | 149 (15.90) | 103 (12.26) | 60 (6.76) |  |  |
| More than seven times a week | 86 (12.13) | 403 (43.01) | 152 (18.10) | 50 (5.64) |  |  |
| Data missing | 25 (3.53) | 18 (1.92) | 36 (4.29) | 34 (3.83) |  |  |
| Other dark-color vegetables |  |  |  |  | 530.301 | < 0.001 |
| Never | 0 (0.00) | 0 (0.00) | 0 (0.00) | 10 (1.13) |  |  |
| Less than once a week | 20 (2.82) | 0 (0.00) | 9 (1.07) | 23 (2.59) |  |  |
| Once a week | 66 (9.31) | 16 (1.71) | 51 (6.07) | 97 (10.94) |  |  |
| Twice to four times a week | 238 (33.57) | 133 (14.19) | 236 (28.10) | 393 (44.31) |  |  |
| Five to six times a week | 170 (23.98) | 200 (21.34) | 206 (24.52) | 199 (22.44) |  |  |
| Seven times a week | 89 (12.55) | 179 (19.10) | 128 (15.24) | 75 (8.46) |  |  |
| More than seven times a week | 108 (15.23) | 399 (42.58) | 188 (22.38) | 76 (8.57) |  |  |
| Data missing | 18 (2.54) | 10 (1.07) | 22 (2.62) | 14 (1.58) |  |  |
| Fresh fruit/vegetable juice |  |  |  |  | 272.279 | < 0.001 |
| Never | 63 (8.89) | 186 (19.85) | 76 (9.05) | 190 (21.42) |  |  |
| Less than once a week | 202 (28.49) | 371 (39.59) | 185 (22.02) | 303 (34.16) |  |  |
| Once a week | 156 (22.00) | 169 (18.04) | 170 (20.24) | 196 (22.10) |  |  |
| Twice to four times a week | 162 (22.85) | 140 (14.94) | 247 (29.40) | 146 (16.46) |  |  |
| Five to six times a week | 36 (5.08) | 24 (2.56) | 62 (7.38) | 29 (3.27) |  |  |
| Seven times a week | 33 (4.65) | 12 (1.28) | 32 (3.81) | 10 (1.13) |  |  |
| More than seven times a week | 43 (6.06) | 13 (1.39) | 55 (6.55) | 5 (0.56) |  |  |
| Data missing | 14 (1.97) | 22 (2.35) | 13 (1.55) | 8 (0.90) |  |  |
| Soybean milk |  |  |  |  | 407.245 | < 0.001 |
| Never | 123 (17.35) | 327 (34.90) | 172 (20.48) | 429 (48.37) |  |  |
| Less than once a week | 232 (32.72) | 366 (39.06) | 251 (29.88) | 317 (35.74) |  |  |
| Once a week | 155 (21.86) | 155 (16.54) | 192 (22.86) | 97 (10.94) |  |  |
| Twice to four times a week | 117 (16.50) | 70 (7.47) | 154 (18.33) | 31 (3.49) |  |  |
| Five to six times a week | 29 (4.09) | 4 (0.43) | 24 (2.86) | 2 (0.23) |  |  |
| Seven times a week | 18 (2.54) | 0 (0.00) | 10 (1.19) | 1 (0.11) |  |  |
| More than seven times a week | 20 (2.82) | 2 (0.21) | 22 (2.62) | 2 (0.23) |  |  |
| Data missing | 15 (2.12) | 13 (1.39) | 15 (1.79) | 8 (0.90) |  |  |
| Milk |  |  |  |  | 192.689 | < 0.001 |
| Never | 22 (3.10) | 26 (2.77) | 5 (0.60) | 32 (3.61) |  |  |
| Less than once a week | 44 (6.21) | 35 (3.74) | 10 (1.19) | 30 (3.38) |  |  |
| Once a week | 54 (7.62) | 26 (2.77) | 14 (1.67) | 41 (4.62) |  |  |
| Twice to four times a week | 153 (21.58) | 132 (14.09) | 61 (7.26) | 125 (14.09) |  |  |
| Five to six times a week | 101 (14.25) | 95 (10.14) | 83 (9.88) | 110 (12.40) |  |  |
| Seven times a week | 97 (13.68) | 126 (13.45) | 140 (16.67) | 120 (13.53) |  |  |
| More than seven times a week | 234 (33.00) | 493 (52.61) | 526 (62.62) | 427 (48.14) |  |  |
| Data missing | 4 (0.56) | 4 (0.43) | 1 (0.12) | 2 (0.23) |  |  |
| Yogurt or other dairy products |  |  |  |  | 162.451 | < 0.001 |
| Never | 15 (2.12) | 16 (1.71) | 5 (0.60) | 13 (1.47) |  |  |
| Less than once a week | 38 (5.36) | 33 (3.52) | 6 (0.71) | 35 (3.95) |  |  |
| Once a week | 47 (6.63) | 38 (4.06) | 23 (2.74) | 62 (6.99) |  |  |
| Twice to four times a week | 209 (29.48) | 194 (20.70) | 133 (15.83) | 238 (26.83) |  |  |
| Five to six times a week | 139 (19.61) | 148 (15.80) | 156 (18.57) | 177 (19.95) |  |  |
| Seven times a week | 117 (16.50) | 156 (16.65) | 179 (21.31) | 142 (16.01) |  |  |
| More than seven times a week | 142 (20.03) | 348 (37.14) | 333 (39.64) | 214 (24.13) |  |  |
| Data missing | 2 (0.28) | 4 (0.43) | 5 (0.60) | 6 (0.68) |  |  |
| Flavored milk drinks |  |  |  |  | 430.035 | < 0.001 |
| Never | 130 (18.34) | 539 (57.52) | 473 (56.31) | 392 (44.19) |  |  |
| Less than once a week | 213 (30.04) | 262 (27.96) | 203 (24.17) | 266 (29.99) |  |  |
| Once a week | 114 (16.08) | 53 (5.66) | 71 (8.45) | 82 (9.24) |  |  |
| Twice to four times a week | 131 (18.48) | 44 (4.70) | 43 (5.12) | 81 (9.13) |  |  |
| Five to six times a week | 42 (5.92) | 8 (0.85) | 11 (1.31) | 27 (3.04) |  |  |
| Seven times a week | 34 (4.80) | 6 (0.64) | 6 (0.71) | 12 (1.35) |  |  |
| More than seven times a week | 33 (4.65) | 11 (1.17) | 9 (1.07) | 13 (1.47) |  |  |
| Data missing | 12 (1.69) | 14 (1.49) | 24 (2.86) | 14 (1.58) |  |  |
| Carbonated drinks |  |  |  |  | 528.948 | < 0.001 |
| Never | 305 (43.02) | 764 (81.54) | 745 (88.69) | 665 (74.97) |  |  |
| Less than once a week | 239 (33.71) | 141 (15.05) | 76 (9.05) | 152 (17.14) |  |  |
| Once a week | 80 (11.28) | 20 (2.13) | 4 (0.48) | 38 (4.28) |  |  |
| Twice to four times a week | 54 (7.62) | 5 (0.53) | 2 (0.24) | 18 (2.03) |  |  |
| Five to six times a week | 14 (1.97) | 1 (0.11) | 0 (0.00) | 7 (0.79) |  |  |
| Seven times a week | 4 (0.56) | 0 (0.00) | 0 (0.00) | 1 (0.11) |  |  |
| More than seven times a week | 8 (1.13) | 1 (0.11) | 0 (0.00) | 1 (0.11) |  |  |
| Data missing | 5 (0.71) | 5 (0.53) | 13 (1.55) | 5 (0.56) |  |  |
| Flavored fruit/vegetable drinks |  |  |  |  | 422.544 | < 0.001 |
| Never | 144 (20.31) | 547 (58.38) | 525 (62.50) | 364 (41.04) |  |  |
| Less than once a week | 309 (43.58) | 305 (32.55) | 222 (26.43) | 336 (37.88) |  |  |
| Once a week | 123 (17.35) | 54 (5.76) | 57 (6.79) | 109 (12.29) |  |  |
| Twice to four times a week | 85 (11.99) | 27 (2.88) | 23 (2.74) | 61 (6.88) |  |  |
| Five to six times a week | 19 (2.68) | 1 (0.11) | 0 (0.00) | 6 (0.68) |  |  |
| Seven times a week | 14 (1.97) | 0 (0.00) | 3 (0.36) | 2 (0.23) |  |  |
| More than seven times a week | 12 (1.69) | 0 (0.00) | 3 (0.36) | 2 (0.23) |  |  |
| Data missing | 3 (0.42) | 3 (0.32) | 7 (0.83) | 7 (0.79) |  |  |
| Tea drinks |  |  |  |  | 503.263 | < 0.001 |
| Never | 365 (51.48) | 824 (87.94) | 772 (91.90) | 721 (81.29) |  |  |
| Less than once a week | 200 (28.21) | 86 (9.18) | 46 (5.48) | 121 (13.64) |  |  |
| Once a week | 65 (9.17) | 15 (1.60) | 5 (0.60) | 27 (3.04) |  |  |
| Twice to four times a week | 43 (6.06) | 4 (0.43) | 1 (0.12) | 10 (1.13) |  |  |
| Five to six times a week | 7 (0.99) | 0 (0.00) | 0 (0.00) | 0 (0.00) |  |  |
| Seven times a week | 3 (0.42) | 0 (0.00) | 0 (0.00) | 2 (0.23) |  |  |
| More than seven times a week | 11 (1.55) | 0 (0.00) | 0 (0.00) | 0 (0.00) |  |  |
| Data missing | 15 (2.12) | 8 (0.85) | 16 (1.90) | 6 (0.68) |  |  |
| Plant-protein drinks |  |  |  |  | 386.514 | < 0.001 |
| Never | 196 (27.64) | 658 (70.22) | 475 (56.55) | 546 (61.56) |  |  |
| Less than once a week | 310 (43.72) | 233 (24.87) | 269 (32.02) | 269 (30.33) |  |  |
| Once a week | 111 (15.66) | 30 (3.20) | 54 (6.43) | 51 (5.75) |  |  |
| Twice to four times a week | 49 (6.91) | 11 (1.17) | 23 (2.74) | 14 (1.58) |  |  |
| Five to six times a week | 19 (2.68) | 0 (0.00) | 5 (0.60) | 0 (0.00) |  |  |
| Seven times a week | 6 (0.85) | 0 (0.00) | 2 (0.24) | 1 (0.11) |  |  |
| More than seven times a week | 10 (1.41) | 0 (0.00) | 1 (0.12) | 0 (0.00) |  |  |
| Data missing | 8 (1.13) | 5 (0.53) | 11 (1.31) | 6 (0.68) |  |  |
| Sweets |  |  |  |  | 645.979 | < 0.001 |
| Never | 23 (3.24) | 51 (5.44) | 32 (3.81) | 4 (0.45) |  |  |
| Less than once a week | 153 (21.58) | 284 (30.31) | 212 (25.24) | 24 (2.71) |  |  |
| Once a week | 194 (27.36) | 226 (24.12) | 198 (23.57) | 86 (9.70) |  |  |
| Twice to four times a week | 242 (34.13) | 298 (31.80) | 300 (35.71) | 404 (45.55) |  |  |
| Five to six times a week | 54 (7.62) | 39 (4.16) | 67 (7.98) | 178 (20.07) |  |  |
| Seven times a week | 21 (2.96) | 21 (2.24) | 17 (2.02) | 83 (9.36) |  |  |
| More than seven times a week | 17 (2.40) | 14 (1.49) | 11 (1.31) | 103 (11.61) |  |  |
| Data missing | 5 (0.71) | 4 (0.43) | 3 (0.36) | 5 (0.56) |  |  |
| Pastries |  |  |  |  | 517.790 | < 0.001 |
| Never | 29 (4.09) | 39 (4.16) | 12 (1.43) | 4 (0.45) |  |  |
| Less than once a week | 209 (29.48) | 334 (35.65) | 155 (18.45) | 59 (6.65) |  |  |
| Once a week | 231 (32.58) | 282 (30.10) | 240 (28.57) | 186 (20.97) |  |  |
| Twice to four times a week | 193 (27.22) | 247 (26.36) | 332 (39.52) | 416 (46.90) |  |  |
| Five to six times a week | 30 (4.23) | 23 (2.45) | 68 (8.10) | 130 (14.66) |  |  |
| Seven times a week | 3 (0.42) | 4 (0.43) | 13 (1.55) | 57 (6.43) |  |  |
| More than seven times a week | 9 (1.27) | 0 (0.00) | 10 (1.19) | 30 (3.38) |  |  |
| Data missing | 5 (0.71) | 8 (0.85) | 10 (1.19) | 5 (0.56) |  |  |
| Puffed foods |  |  |  |  | 597.006 | < 0.001 |
| Never | 113 (15.94) | 374 (39.91) | 366 (43.57) | 108 (12.18) |  |  |
| Less than once a week | 337 (47.53) | 448 (47.81) | 372 (44.29) | 334 (37.66) |  |  |
| Once a week | 152 (21.44) | 87 (9.28) | 71 (8.45) | 242 (27.28) |  |  |
| Twice to four times a week | 85 (11.99) | 19 (2.03) | 20 (2.38) | 167 (18.83) |  |  |
| Five to six times a week | 7 (0.99) | 0 (0.00) | 0 (0.00) | 18 (2.03) |  |  |
| Seven times a week | 5 (0.71) | 0 (0.00) | 0 (0.00) | 8 (0.90) |  |  |
| More than seven times a week | 3 (0.42) | 0 (0.00) | 0 (0.00) | 3 (0.34) |  |  |
| Data missing | 7 (0.99) | 9 (0.96) | 11 (1.31) | 7 (0.79) |  |  |
| Fried foods |  |  |  |  | 559.410 | < 0.001 |
| Never | 78 (11.00) | 270 (28.82) | 274 (32.62) | 65 (7.33) |  |  |
| Less than once a week | 360 (50.78) | 556 (59.34) | 445 (52.98) | 368 (41.49) |  |  |
| Once a week | 181 (25.53) | 98 (10.46) | 97 (11.55) | 291 (32.81) |  |  |
| Twice to four times a week | 70 (9.87) | 9 (0.96) | 16 (1.90) | 132 (14.88) |  |  |
| Five to six times a week | 6 (0.85) | 0 (0.00) | 0 (0.00) | 13 (1.47) |  |  |
| Seven times a week | 3 (0.42) | 0 (0.00) | 1 (0.12) | 4 (0.45) |  |  |
| More than seven times a week | 2 (0.28) | 0 (0.00) | 0 (0.00) | 2 (0.23) |  |  |
| Data missing | 9 (1.27) | 4 (0.43) | 7 (0.83) | 12 (1.35) |  |  |
| Western fast foods |  |  |  |  | 250.366 | < 0.001 |
| Never | 87 (12.27) | 218 (23.27) | 180 (21.43) | 87 (9.81) |  |  |
| Less than once a week | 406 (57.26) | 616 (65.74) | 486 (57.86) | 443 (49.94) |  |  |
| Once a week | 151 (21.30) | 85 (9.07) | 119 (14.17) | 247 (27.85) |  |  |
| Twice to four times a week | 43 (6.06) | 10 (1.07) | 36 (4.29) | 86 (9.70) |  |  |
| Five to six times a week | 6 (0.85) | 0 (0.00) | 2 (0.24) | 11 (1.24) |  |  |
| Seven times a week | 2 (0.28) | 0 (0.00) | 3 (0.36) | 5 (0.56) |  |  |
| More than seven times a week | 4 (0.56) | 1 (0.11) | 1 (0.12) | 2 (0.23) |  |  |
| Data missing | 10 (1.41) | 7 (0.75) | 13 (1.55) | 6 (0.68) |  |  |
| Nuts |  |  |  |  | 316.876 | < 0.001 |
| Never | 41 (5.78) | 63 (6.72) | 10 (1.19) | 50 (5.64) |  |  |
| Less than once a week | 196 (27.64) | 229 (24.44) | 86 (10.24) | 210 (23.68) |  |  |
| Once a week | 201 (28.35) | 228 (24.33) | 141 (16.79) | 249 (28.07) |  |  |
| Twice to four times a week | 201 (28.35) | 299 (31.91) | 314 (37.38) | 272 (30.67) |  |  |
| Five to six times a week | 46 (6.49) | 69 (7.36) | 142 (16.90) | 68 (7.67) |  |  |
| Seven times a week | 8 (1.13) | 14 (1.49) | 66 (7.86) | 22 (2.48) |  |  |
| More than seven times a week | 8 (1.13) | 24 (2.56) | 74 (8.81) | 13 (1.47) |  |  |
| Data missing | 8 (1.13) | 11 (1.17) | 7 (0.83) | 3 (0.34) |  |  |
| Wheat or wheat foods |  |  |  |  | 577.019 | < 0.001 |
| Never | 100 (14.10) | 167 (17.82) | 19 (2.26) | 157 (17.70) |  |  |
| Less than once a week | 231 (32.58) | 395 (42.16) | 111 (13.21) | 315 (35.51) |  |  |
| Once a week | 192 (27.08) | 239 (25.51) | 225 (26.79) | 242 (27.28) |  |  |
| Twice to four times a week | 119 (16.78) | 119 (12.70) | 297 (35.36) | 132 (14.88) |  |  |
| Five to six times a week | 26 (3.67) | 12 (1.28) | 106 (12.62) | 21 (2.37) |  |  |
| Seven times a week | 14 (1.97) | 0 (0.00) | 44 (5.24) | 6 (0.68) |  |  |
| More than seven times a week | 12 (1.69) | 2 (0.21) | 30 (3.57) | 6 (0.68) |  |  |
| Data missing | 15 (2.12) | 3 (0.32) | 8 (0.95) | 8 (0.90) |  |  |
| Meat or poultry |  |  |  |  | 317.384 | < 0.001 |
| Never | 9 (1.27) | 1 (0.11) | 3 (0.36) | 1 (0.11) |  |  |
| Less than once a week | 30 (4.23) | 6 (0.64) | 13 (1.55) | 10 (1.13) |  |  |
| Once a week | 56 (7.90) | 11 (1.17) | 26 (3.10) | 27 (3.04) |  |  |
| Twice to four times a week | 225 (31.73) | 115 (12.27) | 165 (19.64) | 171 (19.28) |  |  |
| Five to six times a week | 182 (25.67) | 169 (18.04) | 171 (20.36) | 192 (21.65) |  |  |
| Seven times a week | 103 (14.53) | 181 (19.32) | 170 (20.24) | 165 (18.60) |  |  |
| More than seven times a week | 99 (13.96) | 446 (47.60) | 285 (33.93) | 312 (35.17) |  |  |
| Data missing | 5 (0.71) | 8 (0.85) | 7 (0.83) | 9 (1.01) |  |  |
| Fishery products |  |  |  |  | 251.284 | < 0.001 |
| Never | 23 (3.24) | 10 (1.07) | 7 (0.83) | 11 (1.24) |  |  |
| Less than once a week | 94 (13.26) | 66 (7.04) | 26 (3.10) | 85 (9.58) |  |  |
| Once a week | 209 (29.48) | 196 (20.92) | 101 (12.02) | 209 (23.56) |  |  |
| Twice to four times a week | 268 (37.80) | 428 (45.68) | 340 (40.48) | 381 (42.95) |  |  |
| Five to six times a week | 64 (9.03) | 141 (15.05) | 163 (19.40) | 118 (13.30) |  |  |
| Seven times a week | 21 (2.96) | 50 (5.34) | 96 (11.43) | 38 (4.28) |  |  |
| More than seven times a week | 21 (2.96) | 41 (4.38) | 93 (11.07) | 41 (4.62) |  |  |
| Data missing | 9 (1.27) | 5 (0.53) | 14 (1.67) | 4 (0.45) |  |  |
| Other protein-rich foods |  |  |  |  | 385.623 | < 0.001 |
| Never | 6 (0.85) | 1 (0.11) | 0 (0.00) | 7 (0.79) |  |  |
| Less than once a week | 20 (2.82) | 5 (0.53) | 2 (0.24) | 21 (2.37) |  |  |
| Once a week | 87 (12.27) | 25 (2.67) | 18 (2.14) | 51 (5.75) |  |  |
| Twice to four times a week | 280 (39.49) | 150 (16.01) | 148 (17.62) | 244 (27.51) |  |  |
| Five to six times a week | 149 (21.02) | 195 (20.81) | 165 (19.64) | 195 (21.98) |  |  |
| Seven times a week | 85 (11.99) | 205 (21.88) | 210 (25.00) | 161 (18.15) |  |  |
| More than seven times a week | 76 (10.72) | 352 (37.57) | 292 (34.76) | 201 (22.66) |  |  |
| Data missing | 6 (0.85) | 4 (0.43) | 5 (0.60) | 7 (0.79) |  |  |
| Notes: ^a^Coffee drinks and energy drinks or sports drinks were eliminated; ^b^Chi-square values were adjusted for ties. | | | | | | |
